# Reciprocal Relationship Between Calcium Signaling and Circadian Clocks: Implications for Calcium Homeostasis, Clock Function, and Therapeutics

**DOI:** 10.3389/fnmol.2021.666673

**Published:** 2021-05-11

**Authors:** Javier Cavieres-Lepe, John Ewer

**Affiliations:** ^1^Centro Interdisciplinario de Neurociencia de Valparaíso, Instituto de Neurociencias, Universidad de Valparaíso, Valparaíso, Chile; ^2^Programa de Doctorado en Ciencias, Mención Neurociencia, Universidad de Valparaíso, Valparaíso, Chile

**Keywords:** daily rhythms, circadian rhythms, biological clocks, E-box, **Drosophila**, chronomedicine

## Abstract

In animals, circadian clocks impose a daily rhythmicity to many behaviors and physiological processes. At the molecular level, circadian rhythms are driven by intracellular transcriptional/translational feedback loops (TTFL). Interestingly, emerging evidence indicates that they can also be modulated by multiple signaling pathways. Among these, Ca^2+^ signaling plays a key role in regulating the molecular rhythms of clock genes and of the resulting circadian behavior. In addition, the application of *in vivo* imaging approaches has revealed that Ca^2+^ is fundamental to the synchronization of the neuronal networks that make up circadian pacemakers. Conversely, the activity of circadian clocks may influence Ca^2+^ signaling. For instance, several genes that encode Ca^2+^ channels and Ca^2+^-binding proteins display a rhythmic expression, and a disruption of this cycling affects circadian function, underscoring their reciprocal relationship. Here, we review recent advances in our understanding of how Ca^2+^ signaling both modulates and is modulated by circadian clocks, focusing on the regulatory mechanisms described in *Drosophila* and mice. In particular, we examine findings related to the oscillations in intracellular Ca^2+^ levels in circadian pacemakers and how they are regulated by canonical clock genes, neuropeptides, and light stimuli. In addition, we discuss how Ca^2+^ rhythms and their associated signaling pathways modulate clock gene expression at the transcriptional and post-translational levels. We also review evidence based on transcriptomic analyzes that suggests that mammalian Ca^2+^ channels and transporters (e.g., *ryanodine receptor, ip3r, serca, L-* and *T*-type Ca^2+^ channels) as well as Ca^2+^-binding proteins (e.g., *camk, cask*, and *calcineurin*) show rhythmic expression in the central brain clock and in peripheral tissues such as the heart and skeletal muscles. Finally, we discuss how the discovery that Ca^2+^ signaling is regulated by the circadian clock could influence the efficacy of pharmacotherapy and the outcomes of clinical interventions.

## Graphical Abstract

Reciprocal relationship between Ca^2+^ signaling and the circadian clock. In *Drosophila* and mice, circadian clocks impose a daily rhythmicity to Ca^2+^ signaling; and, conversely, Ca^2+^ rhythms and signaling contribute to transmitting daily external signals to the clock TTFL. This bidirectional regulation is critical to the daily rhythmicity of many physiological and behavioral processes.

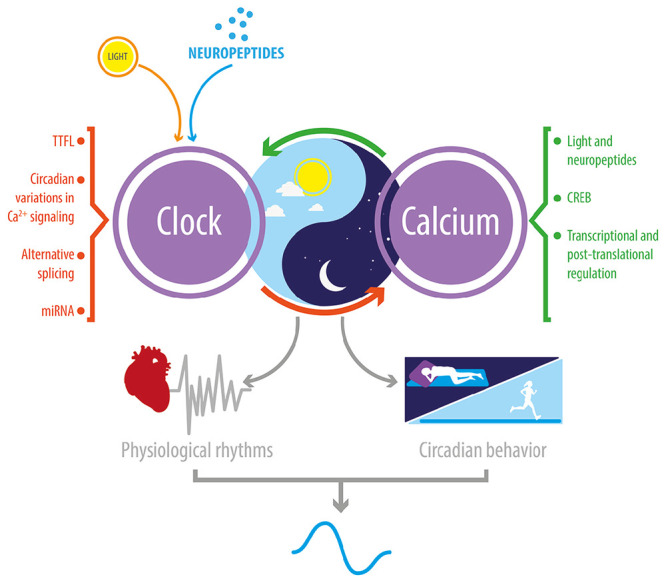

## Introduction

All multicellular animals contain a biological clock that allows them to anticipate the daily changes in the environment, such as the arrival of dawn or dusk. These are endogenous mechanisms that are cell-autonomous and are invariably controlled by highly conserved intracellular transcription-translation feedback loops (TTFL) (Hardin, 2011; Takahashi, 2017). At their core, these TTFLs include the transcription factors, CLOCK and CYCLE (usually called BMAL1 in mammals), which activate the transcription of the *period* (*per*) and either the *timeless* (*tim*) or the *cryptochrome* (*cry*) genes in *Drosophila* and mammals, respectively. In the cytoplasm, the corresponding PER and either TIM (*Drosophila*) or CRY (mammals) proteins dimerize, re-enter the nucleus, and inhibit the activity of CLOCK-CYCLE, thereby preventing their own expression. The exact functioning of this core TTFL is, in addition, modified by a growing number of intracellular factors, which include additional secondary TTFLs, kinases and phosphatases that regulate the stability of PER and TIM (CRY) proteins, as well as chromatin and chromatin-modifying complexes, all of which contribute to producing a molecular rhythm with near-24 h periodicity. Circadian clocks are classified according to where they reside: central oscillators are located in the brain whereas peripheral oscillators can be housed in a wide variety of tissues, where they impose a daily rhythmicity to the physiology of each organ (Mohawk et al., 2012). Yet, the different cells that make up the central oscillators must coordinate their activity and must also be entrainable by the daily environmental signals that synchronize the organism’s pattern of activity to the appropriate time of day (usually light, but for ectothermal animals can also be temperature). In addition, peripheral clocks are coordinated with the central clock to produce a unitary biological time for the organism. And, finally, the activity of clocks can in turn be modified by the organism’s physiology and/or behavior (e.g., through feeding, Damiola et al., 2000). At the systems level, the entrainment of central and peripheral clocks, the coordination between clocks as well as between clocks and their host, occurs via neuronal, endocrine, and paracrine signals (Schibler et al., 2015; King and Sehgal, 2020). These signals are transduced by various signaling pathways that ultimately alter the temporal pattern of gene expression. Here, we review the role of Ca^2+^ in clock function by considering intracellular and extracellular signals that regulate Ca^2+^ levels in circadian clocks (see section “Ca^2+^ Rhythms in Biological Clocks Are Regulated by Intracellular and Extracellular Signals”). In addition, we discuss the mechanism by which Ca^2+^ signaling affects clock function (see section “Effects of Ca^2+^ Signaling on the Expression of Clock Genes and Rhythmic Behavior”), and, in turn, how clock function affects Ca^2+^ signaling (see section “Circadian Regulation of Ca^2+^ Associated Proteins”). Finally, as a result of our growing appreciation of the importance of clocks in health and disease, we discuss how our knowledge of the reciprocal relationship between clocks and Ca^2+^ signaling impacts the effectiveness of drugs and is relevant to the development of improved therapeutics (see section “Ca^2+^ Signaling and Clocks: Implications for Diseases and Chronomedicine”).

## Ca^2+^ Rhythms in Biological Clocks Are Regulated by Intracellular and Extracellular Signals

In animals, intracellular Ca^2+^ levels ([Ca^2+^]_i_) are essential for the proper functioning of many cellular and physiological processes, including circadian rhythms. For example, in the case of pacemakers housed within neuronal tissues, fluctuations in [Ca^2+^]_i_ have been associated with the circadian control of neuropeptide release, the expression of clock genes, synaptic plasticity, and the periodicity of rhythmic behaviors (Lundkvist et al., 2005; Harrisingh et al., 2007; Depetris-Chauvin et al., 2011). For this reason, an important area of research in chronobiology is devoted to identifying changes in [Ca^2+^]_i_ that occur in circadian pacemaker neurons and understanding their origin. Interestingly, daily Ca^2+^ variations occur in circadian oscillators of rodents (Colwell, 2000; Ikeda et al., 2003; Enoki et al., 2012, 2017; Brancaccio et al., 2013; Noguchi et al., 2017), flies (Liang et al., 2016; Guo et al., 2017), and mollusks (Colwell et al., 1994).

### Neuropeptide-Mediated Ca^2+^ Rhythms

In mice, Ca^2+^ rhythms have been described in neurons and astrocytes of the central mammalian clock, which is located in the suprachiasmatic nucleus (SCN) (Ikeda et al., 2003; Brancaccio et al., 2017). Remarkably, they are not uniform over the entire nucleus. Indeed, the phase of Ca^2+^ oscillations in dorsal regions are advanced relative to those in the ventral zone, and this spatial organization is highly dependent on neuronal network properties within the SCN (Enoki et al., 2012; Brancaccio et al., 2013). In addition, this difference in Ca^2+^ phase is abolished when the intercellular synchronization within the SCN is weak (Enoki et al., 2017), suggesting that diffusible factors such as the neuropeptides, arginine vasopressin (AVP) or vasoactive intestinal peptide (VIP), which are enriched in dorsal and ventral regions of SCN, respectively (see Figure 4 from Enoki et al., 2017), are relevant for maintaining the phase difference between Ca^2+^ oscillations in the dorsal vs. ventral SCN. Similarly, in *Drosophila*, where daily locomotor activity shows a bi-modal pattern, the central clock neurons that control the morning and the evening peaks of activity express different Ca^2+^ phases (Liang et al., 2016). In particular, neurons that regulate the morning activity (the so-called M-cells) exhibit an advanced Ca^2+^ phase relative to circadian pacemaker neurons that control the evening activity (the so-called E-cells) (see Figure 2 in Kozlov and Nagoshi, 2019). Remarkably, inhibiting signaling mediated by pigment dispersing factor (PDF), a neuropeptide analogous to mammalian VIP, causes the phases of Ca^2+^ oscillations in M and E cells to be synchronous (Liang et al., 2016) and the pattern of behavior to be unimodal or arrhythmic (Hyun et al., 2005). In addition, in *Drosophila*, [Ca^2+^]_i_ varies during the course of the day in the prothoracic gland (PG), a peripheral clock that regulates the circadian rhythm of adult eclosion (Morioka et al., 2012; Palacios-Munoz and Ewer, 2018). A study carried out in organotypic PG cultures shows that the phase of the Ca^2+^ oscillations is set by inputs from the brain (Morioka et al., 2012), which are probably mediated by prothoracicotropic hormone, a neuropeptide that transmits time information from the central clock to the PG (Selcho et al., 2017). Of note, Ca^2+^ plays a critical role in the synthesis and release of the molting hormone, ecdysone, from the PG (Huang et al., 2008; Smith et al., 2012; Yamanaka et al., 2015), suggesting that fluctuations in [Ca^2+^]_i_ due to clock activity could produce a daily rhythm in hemolymph ecdysone titers and, consequently, be relevant to the circadian control of adult emergence. Nevertheless, although such oscillations occur in some insects (Ampleford and Steel, 1985), they have not been detected in *Drosophila.*

### Clock Genes and Ca^2+^ Oscillations

Although neuropeptides can set the phase of Ca^2+^ oscillations in biological clocks, these extracellular signals are not the only relevant ones for regulating Ca^2+^ rhythms. In mice, an early study using FURA-2, a synthetic fluorescent Ca^2+^-sensitive dye, showed that Ca^2+^ oscillations are completely abolished by tetrodotoxin (TTX) treatments, suggesting that neuronal firing of SCN neurons is essential for maintaining these rhythms (Colwell, 2000). However, FURA-2 does not allow for the long-term measurement of Ca^2+^ oscillations because it is eventually cleared from the cytoplasm. In contrast to these results, studies that have expressed genetically encoded Ca^2+^ sensors such as GCaMP or Cameleon in the SCN have shown that Ca^2+^ rhythms are only partially reduced in the absence of neuronal firing, suggesting that intracellular signaling and possibly also non-action potential dependent signaling (e.g., Hablitz et al., 2020) also plays a role in regulating the oscillations of [Ca^2+^]_i_ (Enoki et al., 2012; Noguchi et al., 2017). Intracellular pathways could be associated with TTFLs of clock genes. Indeed, in mammals, overexpression of a dominant-negative allele of BMAL1, a transcription factor and core element of the clock, inhibits Ca^2+^ rhythms in the SCN (Ikeda and Ikeda, 2014). Similarly, in *Drosophila* a null allele of the *period* gene, which is also a core component of the clock, reduces the rhythm and coherence of Ca^2+^ oscillations in central clock neurons (Liang et al., 2016), and similar results have been described for the peripheral clock housed in the prothoracic gland (Palacios-Munoz and Ewer, 2018).

### Photic Inputs Set the Phase of Ca^2+^ Oscillations

A third regulator of intracellular Ca^2+^ rhythms is photic inputs. Light is the most potent signal for phase shifting and for entraining circadian rhythms of behavior. In the mammalian SCN, light causes a time-dependent shift in the phase of the spontaneous Ca^2+^ oscillations exhibited by neurons that produce VIP, which are critical for propagating photic inputs through the SCN as well as for resetting the daily rhythms (Jones et al., 2018). Consistent with this, the behavioral rhythms of mice lacking calbindin (a cytosolic Ca^2+^-buffering protein) display increased phase delays when they receive a light stimulus in the early part of the night (Stadler et al., 2010). Interestingly, in rodents, photic inputs also regulate Ca^2+^ rhythms using intracellular stores (Ding et al., 1998; Ikeda et al., 2003; Aguilar-Roblero et al., 2007). Indeed, blockers of the ryanodine receptor impair Ca^2+^ oscillations in organotypic cultures of rat SCN (Ding et al., 1998), suggesting that the release of Ca^2+^ from the endoplasmic reticulum is critical for the regulation of the rhythm of [Ca^2+^]_i_ in the mammalian central clock. On other hand, in *Drosophila*, photic inputs set the phase of Ca^2+^ oscillations in central clock neurons through two pathways: they either act through the visual system via PDF, or they act directly on the circadian pacemaker through the internal photoreceptor, CRYPTOCROME (CRY), to set the phase of a group of E-cell neurons (Liang et al., 2017). By contrast, in the PG, CRY-dependent photoreception inhibits Ca^2+^ activity and this effect is abolished when the brain-PG complex is treated with TTX, suggesting that in this peripheral oscillator the actions of light are mediated by neuronal pathways from the brain (Morioka et al., 2012). In insects, light can also penetrate the translucid exoskeleton and entrain peripheral clocks; however, whether these photic inputs can directly affect the Ca^2+^ rhythms of pacemaker neurons is currently unknown.

**FIGURE 1:**
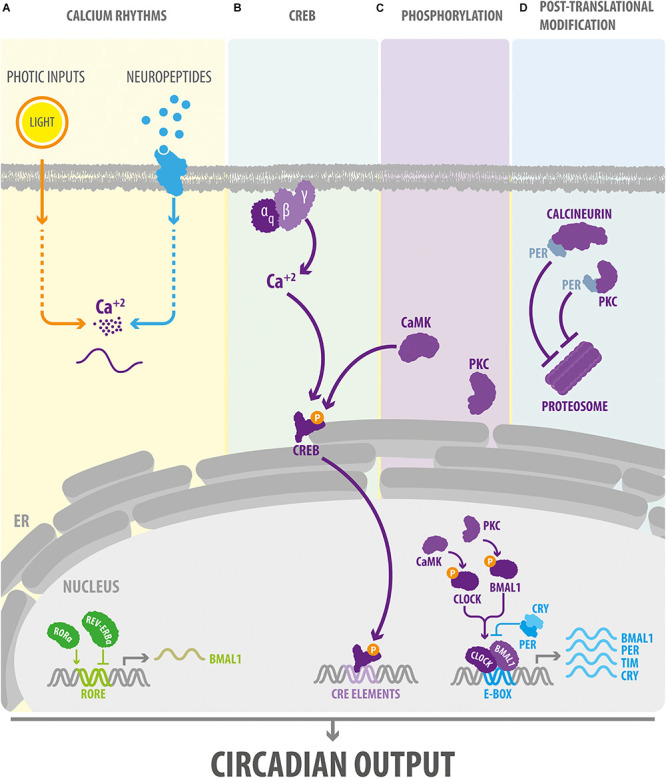
Ca^2+^ signaling modulates the core components of the circadian clock. **(A)** In *Drosophila* and mammals, intracellular cytosolic Ca^2+^ rhythms are regulated by external signals such as light stimuli and neuropeptides. **(B)** In the SCN, external signals may affect the TTFL via the G_*q*_-Ca^2+^ pathway. In turn, Ca^2+^ signaling via CREB and the regulation of the phosphorylation state **(C)** and proteasomal degradation **(D)** of clock proteins, orchestrate the effect of Ca^2+^ on the circadian clock in *Drosophila* and mammals. BMAL1, Brain and Muscle ARNT-Like 1, ortholog of *Drosophila Cycle* gene; CaMK, Ca^2+^/calmodulin-dependent protein kinase. CLOCK, Circadian Locomotor Output Cycles Kaput; CREB, cAMP response element-binding protein; CRE elements, cAMP response elements; CRY, cryptochrome; E-box, circadian enhancer box; ER, endoplasmic reticulum; PER, PERIOD protein; PKC, protein kinase C; ROR-α, retinoid-related orphan receptor alpha; TIM, TIMELESS protein.

**FIGURE 2:**
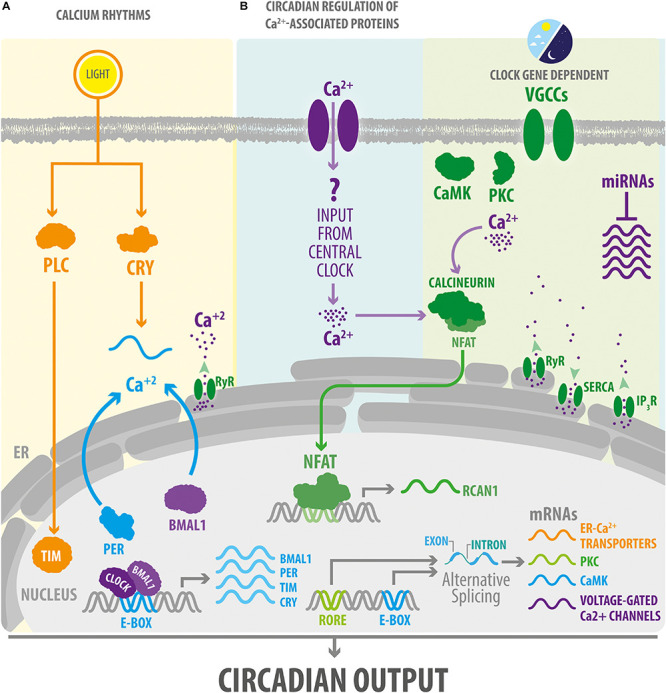
Circadian clocks impose a daily rhythm on intracellular Ca^2+^ signaling. **(A)** Photic inputs via CRY or other signaling molecules such as PLC cause a phase-shift in the expression of molecular clock components in the mammalian and *Drosophila* central clocks. In turn, TTFL regulate cytosolic Ca^2+^ rhythms. In SCN neurons, circadian oscillations of Ca^2+^ levels are also dependent on the mobilization of Ca^2+^ from the endoplasmic reticulum. **(B)** Circadian clocks impose a daily rhythm of expression to a large number of components of the Ca^2+^ signaling pathway by acting at transcriptional or post-transcriptional levels (including by regulating alternative splicing in *Drosophila* and microRNA in mice). In addition, in mice, the Ca^2+^/calcineurin/NFAT pathway exhibits a rhythmic activity in peripheral clocks, such as the one present in skeletal muscle or heart, which is probably mediated by inputs from the central clock. IP_3_R, inositol 1,4,5-triphosphate Receptor; NFAT, nuclear factor of activated T-cells; RORE, ROR response elements. RCAN1, regulator of calcineurin 1. RyR, ryanodine receptor. SERCA, sarcoplasmic/endoplasmic reticulum Ca^2+^ ATPase. See Figure 1 for other abbreviations.

Overall, this evidence suggests that Ca^2+^ rhythms in circadian clocks are regulated by both intracellular and extracellular signals. In addition, extracellular signals from neuropeptides and photic inputs can set the phase of these rhythms and coordinate the relative timing of the Ca^2+^ oscillations (Figure 1A), whereas TTFLs, and probably also intracellular Ca^2+^ stores, can contribute to generate Ca^2+^ oscillations in circadian clocks (Figure 2A). This model is based on the proposal that the central clock is comprised of interconnected autonomous circadian oscillators whose emerging network properties reinforce their circadian rhythmicity, synchronizing their oscillations, and adjusting them to the day-night cycles (Welsh et al., 2010).

## Effects of Ca^2+^ Signaling on the Expression of Clock Genes and Rhythmic Behavior

In multicellular organisms, a variety of molecules are critical for sustaining circadian behavior. In *Drosophila* and mammals, transcription factors, microRNAs, and protein kinases, control the proper functioning of biological clocks (Patke et al., 2020). Interestingly, Ca^2+^ signaling is also involved in driving rhythmic behaviors and rhythms of gene expression. In *Drosophila*, genetic manipulations that decrease Ca^2+^ levels or reduce the expression of proteins such as Ca^2+^/calmodulin-dependent protein kinase II (CaMKII) in circadian pacemaker neurons, lengthen the periodicity of the circadian rhythms of locomotor activity and of adult eclosion (Harrisingh et al., 2007; Palacios-Munoz and Ewer, 2018). Similarly, a mouse bearing a mutation in the CaMKII gene that abolishes all kinase activity exhibits a longer free-running period of locomotor activity and a desynchronization between the molecular rhythms of the left and right nuclei of the SCN (Kon et al., 2014). In terms of gene expression, buffering intracellular Ca^2+^ abolishes *Per1* oscillations in cultured SCN slices, and voltage-gated Ca^2+^ channel antagonists dampen the rhythm of *Per2* and *Bmal1* in an SCN cell line (Lundkvist et al., 2005; Nahm et al., 2005). Likewise, in the peripheral clock of the rat liver, lowering extracellular [Ca^2+^] abolishes *Per1* rhythmicity (Lundkvist et al., 2005). Remarkably, blocking Ca^2+^ flux mediated by the 1,4,5-trisphosphate receptor (IP_3_R) and the sarco/endoplasmic reticulum Ca^2+^ ATPase (SERCA) in rat liver explants lengthens the period of *Per1-luc* oscillations (Baez-Ruiz and Diaz-Munoz, 2011), which provides additional evidence that intracellular Ca^2+^ stores play a key role in the generation of circadian rhythms.

The growing evidence of the role of Ca^2+^ in the control of the clock genes expression raises the question of how Ca^2+^ signaling would influence the TTFL. To date, three mechanism has been proposed: (a) regulation via Ca^2+^/cAMP-responsive elements (CREs); and by modifying the phosphorylation state of clock proteins that affect the functioning of TTFLs at (b) the transcriptional or (c) the post-translational level.

### Ca^2+^/cAMP-Responsive Elements

cAMP-responsive element is a sequence that binds CRE-binding protein (CREB) and is present within the promoter region of several mammalian clock genes (Zhang et al., 2005). In flies and mice, CREB induces changes in clock gene expression in response to light and contributes to the synchronization of activity among pacemaker cells (Ginty et al., 1993; Welsh et al., 2010; Tanenhaus et al., 2012). Several findings suggest that CREB is critical for integrating the signaling mediated by second messengers, such as Ca^2+^, into the rhythm of expression of clock genes. For instance, in the hamster SCN, the phase-shifts in *Per1* and *Per2* induced by light are mediated by CaMKII (Yokota et al., 2001), which can stimulate CRE-promotor activity through the phosphorylation of CREB (Figure 1B) (Nomura et al., 2003). In addition to actions mediated through CaMK signaling, manipulations of electrical activity in neurons reveals a close relationship between Ca^2+^-induced changes in CREB expression and alterations in TTFL. In *Drosophila*, for example, the hyperexcitation of a cluster of clock neurons triggers a morning-like transcriptome profile whereas their hyperpolarization induces an evening-like transcription state (Mizrak et al., 2012). Many of the genes differentially expressed in response to electrical activity code for K^+^ channels and proteins that are part of the PKC, PI_3_K, and MAPK signaling pathways, and are enriched in CRE elements in their promotor region. In addition, manipulating the state of excitability of clock neurons regulates CREB expression, respectively, increasing or decreasing its levels in response to depolarization or hyperpolarization at night (Mizrak et al., 2012). These findings suggest that, in *Drosophila*, the electrical state of clock neurons imposes a daily rhythm to the transcriptional profile via CREB. On other hand, assays using a CRE-luciferase reporter system have shown that CRE-mediated transcription exhibits a circadian rhythmicity in neurons and glia in multiple areas of the *Drosophila* brain (Tanenhaus et al., 2012); in the mouse SCN, this oscillation is highly dependent on the Gq-Ca^2+^ axis (Figure 1B) (Brancaccio et al., 2013). In particular, the Ca^2+^ peak occurs earlier than the CRE-luc peak, which in turn, precedes the peaks in *Per1-luc* and *Per2*-luc activity (see in Figure 4 from Enoki et al., 2017). Remarkably, the activation of the G_*q*_-dependent pathways using DREADDs (designer receptor exclusively activated by designer drug) reorganizes this hierarchical organization altering Ca^2+^ rhythms in clocks cells and lengthening the period of CRE-luc and *Per-luc* cycling in the SCN. These effects are not mediated through G_*s*_ or G_i_, indicating that CRE-mediated transcription is exclusively activated through the Gq-Ca^2+^ axis.

### Ca^2+^ Signaling and Transcriptional Changes Mediated by Phosphorylation of Clock Proteins

The phosphorylation state of clock proteins plays a pivotal role in the functioning of circadian clocks. Indeed, mutations in a variety of protein kinase and phosphatases alter the rhythm of circadian behaviors in flies, hamsters, mice, and humans (Reischl and Kramer, 2011). At the molecular level, kinase activity can regulate the transcription levels of canonical clocks genes, their accumulation in the nucleus, their ability to bind other proteins, and their stability (Gallego and Virshup, 2007). Interestingly, Ca^2+^ signaling has been implicated in the control of the phosphorylation state of clock proteins (Figure 1C). For instance, protein kinase C (PKC) and phospholipase C are critical to light-induced clock resetting in mice (Jakubcakova et al., 2007) and *Drosophila* (Saint-Charles et al., 2016; Ogueta et al., 2018, 2020), respectively. In the mouse SCN, the adaptation to photic stimuli is highly dependent on the phosphorylation state of chromatin modifiers mediated by PKC. In particular, PKCα phosphorylates a lysine-specific demethylase 1 (LSD1) that controls the phase resetting of pacemaker cells and the circadian control of locomotor activity in mice (Nam et al., 2014). At the molecular level, phosphorylated LSD1 binds the BMAL1/CLOCK complex, which controls the pattern of expression of clock genes that contain an E-box sequence (a circadian transcriptional enhancer) in their promotor region (Nam et al., 2014). In addition, in a variety of mammalian cell types, PKCα can act autonomously on core clock proteins, for instance by phosphorylating BMAL1, and this activity is enhanced by RACK1 (receptor for activated C kinase-1), a signaling protein that recruits PKC to its substrates (Robles et al., 2010). The PKCα/RACK-1 complex is recruited to BMAL1 in a circadian manner in central and peripheral mammalian pacemakers. In a fibroblast cell line, the PKCα/RACK1 complex controls the circadian period by acting as a negative regulator of BMAL1-CLOCK transcriptional activity, which supports the idea that PKC plays a key role in regulating mammalian circadian clocks (Robles et al., 2010). CaMKII is another protein associated with Ca^2+^ signaling that directly phosphorylates core clock proteins in the mouse SCN. Indeed, in pacemaker cells of this brain area, this protein kinase phosphorylates CLOCK in a circadian manner by promoting BMAL1-CLOCK heteromerization and enhancing E-box-dependent gene expression including that of *Per1-3* and *Cry1* (Kon et al., 2014). Remarkably, in the mammalian SCN, the pattern of expression of these genes is also altered by calmodulin inhibitors and Ca^2+^ chelators (Kon et al., 2014), indicating that the Ca^2+^/calmodulin/CaMKII-mediated phosphorylation of CLOCK is an important regulator of cell-autonomous clockwork periodicity.

### Ca^2+^ Signaling and Posttranslational Modifications Mediated by the Phosphorylation of Clock Proteins

In the circadian clocks of flies and mammals, the actions mediated through Ca^2+^ modulation of kinase/phosphatase activity are not restricted to the nucleus of pacemaker cells (Figure 1D). For instance, transfection of PKC in mammalian cell lines increases the stability of PER2 and promotes its cytoplasmic localization (Jakubcakova et al., 2007). These effects are independent of the CREB pathway and may involve CK1ε (casein kinase 1 epsilon), which is known to regulate the proteasomal degradation of PER2 and its shuttling between the cytoplasm and the nucleus (Akashi et al., 2002; Eide et al., 2005). On other hand, in *Drosophila*, calcineurin, a Ca^2+^/calmodulin-dependent serine/threonine phosphatase, also controls the stability of clock proteins at the posttranslational level (Kweon et al., 2018). In particular, a null mutant of *sarah*, a calcineurin regulator, reduces the levels of TIM (also a canonical clock protein in *Drosophila*) and PER proteins in head extracts but not those of their respective transcripts. Of note, the effect of calcineurin on clock proteins levels is abolished when the proteasomal machinery is inhibited (Kweon et al., 2018), suggesting that in *Drosophila*, calcineurin regulates the core clock mechanism by protecting PER and TIM from proteasomal degradation.

Collectively, these findings demonstrate that the role of Ca^2+^ signaling is not limited to transducing the actions of external inputs within circadian pacemakers. Instead, studies in mammals and flies show that PKC, CaMK, calmodulin, and calcineurin, are integral components of clocks’ transcriptional and posttranslational feedback loops (Figures 1C,D). How is circadian rhythmicity decoded by these signaling protein? Here, we propose a model in which the oscillations of free Ca^2+^ in the cytoplasm impose a daily rhythm to the activity of its associated proteins (Figure 1). Thus, Ca^2+^ and its signaling pathways may regulate the molecular machinery of pacemaker cells and, consequently, also the periodicity of the resulting circadian behavior.

## Circadian Regulation of Ca^2+^ Associated Proteins

### Rhythmic Expression of Genes Coding for Proteins Associated With Ca^2+^ Pathways

In animals, the core molecular clock machinery directly or indirectly controls the expression of multiple downstream genes involved in the generation of rhythmic cellular process and circadian behaviors. Interestingly, in both mammals and *Drosophila*, a number of genes coding for proteins associated with Ca^2+^ pathways exhibit rhythmic transcription within a variety of biological clocks. For example, microarray and high throughput RNAseq analyses of mouse SCN reveal that genes that code for key proteins in Ca^2+^ signaling pathways such as *camkii*, *pkc-*α, and *calcineurin*, are under circadian control (Table 1) (Panda et al., 2002; Pembroke et al., 2015). These findings are consistent with previous studies that demonstrate that PKC activity shows a daily rhythm in SCN cells lines (Rivera-Bermudez et al., 2003) and that CaMKII is rhythmically expressed in the hamster central pacemaker (Agostino et al., 2004). In mice, canonical endoplasmic reticulum Ca^2+^ transporters (*serca*, *ip_3_r*, and *ryr*) also exhibit circadian regulation at the transcriptional level in the SCN (Table 1). Interestingly, the contribution of the RyR to the phase-shifting mediated by light in the central clock is restricted to the early night (Ding et al., 1998), which in the mouse coincides with the time of peak protein levels in SCN [Table 1 and (Pfeffer et al., 2009)] and, as a result, cytosolic Ca^2+^ reaches high enough levels to phase shift the core molecular clock. Importantly, in mice, the promotor regions of *camkii, pkc*α, *serca, ip_3_r*, and *ryr*, include E-BOX and ROREs motifs (consensus sequences for transcriptional regulation mediated by core clock components) (Table 1), suggesting that the rhythmic oscillation in mRNA levels of these genes may result from a direct regulation by the molecular machinery of the clock. Indeed, a deletion in a promotor region of *ryr2* that includes the E-box sequence, reduces the transcriptional activation induced by the BMAL1-CLOCK complex in a mouse fibroblast cell line (Pfeffer et al., 2009), supporting the idea that the molecular clockwork can directly influence Ca^2+^ signaling.

**TABLE 1:** Circadian regulation of genes coding for proteins associated with Ca^2+^ signaling in mice.

**Gene name**	**Description of the encoded protein**	**E-box^a^**	**ROREs^b^**	**Oscillates in SCN^c^**	**Acrophase**^d^
ip3r	IP3-sensitive Ca2^+^ channel localized to the ER	√ (1169)	√ (1020)	√	Night
pkcα	Protein kinase that can be activated by Ca^2+^	√ (15780)	√ (680)	√	Night
camkiiα	Protein kinase that is regulated by the Ca^2+^/calmodulin complex	√ (3420)	√ (1200)	√	Night
rack1	Intracellular protein receptor for PKC	√ (720)	√ (700)	√	Morning
plcβ-1	Enzyme mediator of signal transduction through Ca^2+^ pathways	X	√ (1260)	X	Night
serca	Ca^2+^ transporter ATPase from cytosol to ER	√ (10.173)	√ (1680)	X	Night
calcineurin	Ca^2+^-dependent protein phosphatase involved signal transduction	√ (4340)	√ (1740)	√	Morning
cask	Protein kinase that regulates signal transduction in multiple pathways including Ca^2+^	√ (7340)	√ (2280)	√	Morning
cacna1c	Subunit of L-type voltage-dependent Ca^2+^ channel	√ (6400)	√ (420)	X	Night
cacna1g	Subunit of T-type voltage-dependent Ca^2+^ channel	√ (3610)	√ (3680)	√	Evening
ryr3	Ca^2+^ release channel in the ER in response to depolarization of the cell expressed in neurons	√ (13140)	√ (2200)	√	Night

*^a^Number of base pairs upstream of the transcription start site where the E-BOX sequence (CACGTG) (Hao et al., 1997) is found.*

*^b^Number of base pairs upstream of the transcription start site where the ROR response sequence (AGGTCA) (Preitner et al., 2002) is located.*

*^c^Circadian oscillation in the transcript levels of genes in SCN according to database generated using HTseq and DESeq2 (http://wgpembroke.com/shiny/SCNseq/) (Pembroke et al., 2015).*

*^d^“Morning” refers to ZT0-ZT6; “evening” to ZT6-ZT12; and “night” to ZT12-24.*

In mammals, the central clock can regulate Ca^2+^ influx from the extracellular milieu via voltage-gated Ca^2+^ channels (VGCCs). Indeed, in the SCN, some VGCCs subunits mediate the phase shifting induced by photic inputs as well as the daily changes in conductance (Colwell, 2011). In addition, L-type, T-type, and P/Q type VGCCs, are under circadian control at the transcriptional level in the rat SCN (Nahm et al., 2005). Interestingly, the rhythmic expression of L-type Ca^2+^ channels, the most abundant VGCCs in the SCN, is regulated by the circadian clock component, REV-ERBα (Schmutz et al., 2014). In particular, in mouse hypothalamic tissues (including the SCN), REV-ERBα binds to the promotor region of an L-type Ca^2+^ channel (*Cacna1c*) at ROREs and direct mutagenesis of this sequence abolishes the oscillation of *Cacna1c* mRNA levels, indicating that REV-ERBα plays a critical role in this circadian regulation. Of note, this mechanism is not the only one that has been associated with the rhythmic control of expression of L-type VGGCs. Indeed, in chicken cone photoreceptors, L-type-VGCCα1Cs display a diurnal rhythm of expression, which is controlled at the posttranscriptional level by microRNAs (Shi et al., 2009). In particular, in the retina, microRNA-26a exhibits a rhythmic expression and binds to the untranslated region of an *L-type-VGGC*α*1C* during the subjective day, thereby imposing a rhythm to the translation of this Ca^2+^ channel. Although there is evidence that microRNAs participate in the circadian control of transcription in the mammalian central clock (Cheng et al., 2007), it is still unknown whether they act at the posttranscriptional level on genes encoding proteins associated with Ca^2+^ signaling.

Another interesting case of circadian transcriptional regulation involving Ca^2+^ pathway proteins occurs in the *Drosophila* central clock. The adult fly brain has around 150 clock neurons per hemisphere, which are classified based on their anatomical location (Helfrich-Forster et al., 2007). These include so-called lateral (LN) and dorsal (DN) clusters of neurons (each of which can be further classified into subclusters), and a recent study using RNA-seq showed that each subcluster exhibits a different transcriptional profile (Abruzzi et al., 2017). Interestingly, a number of genes that are differentially expressed encode proteins of the Ca^2+^ pathway. For instance, dorsal lateral pacemaker neurons (LNds) (but not ventral lateral pacemaker neurons, LNvs), display a rhythmic expression in members of the PKC pathway (*norpA, pkc53E, pkc98-E*, and *pkc*α), and in genes encoding Ca^2+^ channels (*cacophony*, Ca^2 +^ α 1D) and genes involved in Ca^2+^ signaling (*CaMK*, *calcineurin*) (Table 2). Many of these genes contain E-box or RORE sequences in their promotor region, suggesting that the circadian transcriptional control is directly mediated by core clock components (Table 2). However, although LNd and DN1 neurons exhibit a similar circadian pattern of expression of genes encoding Ca^2+^-associated proteins, the spatiotemporal patterns of Ca^2+^ activity of LNds vs. DN1s are quite different (Liang et al., 2016) (see Figure 2 in Kozlov and Nagoshi, 2019), suggesting the existence of additional levels of regulation. What could be the underlying molecular mechanism? Wang et al. (2018) evaluated the alternative pre-mRNA splicing in *Drosophila* clock neurons, which is recognized as a major mechanism used to diversify the neuronal proteome. Combining RNAseq and computational methods, these authors quantified and categorized pre-mRNAs in pacemaker cells observing that each subgroup of pacemaker neurons possesses a unique alternative splicing profile. Of note, many transcripts of the Ca^2+^-calmodulin-dependent family of protein kinases, PKC signaling, and Ca^2+^ channels, were differentially enriched in DN1 vs. LNd pacemaker neurons, suggesting that posttranscriptional regulation may be critical for differentiating the roles of each circadian neuronal cluster in order to produce a functional circadian clock.

**TABLE 2:** Circadian regulation of genes coding for proteins associated with Ca^2+^ signaling in *Drosophila*.

**Gene name**	**Description of the encoded protein**	**E-box^a^**	**RevRE^b^**	**Oscillates in central clock^c^**
ip3r	IP3-sensitive Ca^2+^ channel localized to the ER	√ (120)	√ (4080)	Not described
pkc53E	Protein kinase that can be activated by Ca^2+^	√ (4400)	√ (10920)	√ (LNd)
norpA	Protein kinase that can be activated by Ca^2+^ associated with phototransduction	√ (7900)	√ (3840)	√ (LNd)
camkii	Protein kinase that is regulated by the Ca^2+/^calmodulin complex	X	X	√ (LNd)
rack1	Intracellular protein receptor for PKC	X	X	Not described
plc21c	Enzyme mediator of signal transduction through Ca^2+^ pathways	√ (1020)	√ (900)	√ (LNv)
serca	Ca^2+^ transporter ATPase from cytosol to ER	√ (480)	X	Not described
calcineurin B	Ca^2+^-dependent protein phosphatase involved signal transduction	X	X	√ (LNd/DN1)
cask	Protein kinase that regulates signal transduction in multiple pathways including Ca^2+^	√ (7800)	√ (16680)	√ (LNd/DN1)
Ca^2+^α-1D	α subunit of an L-type voltage-gated Ca^2+^ channel expressed in neurons	X	√ (2700)	Not described
cacophony	Subunit of a voltage-gated Ca^2+^ channel located at presynaptic active zones	X	√ (1320)	√ (LNd)
ryr	Ca^2+^ release channel in the ER in response to depolarization of the cell	√ (2400)	X	Not described

*^a^Number of base pairs upstream of the transcription start site where the E-BOX sequence (CACGTG) (Hao et al., 1997) is found.*

*^b^Number of base pairs upstream of the transcription start site where the RevRE sequence (AGGTCA) (Preitner et al., 2002) is located.*

*^c^Circadian oscillation in levels of transcripts for genes in the central clock (specific neuronal group is indicated in parenthesis) according to database generated using RNAseq (Abruzzi et al., 2017). LN, lateral neuron cluster (v, ventral; d, dorsal); DN1, dorsal neuron cluster 1.*

### Skeletal Muscle and Heart, Two Peripherals Clocks Displaying Rhythmic Changes in the Transcription of Elements of the Ca^2+^ Signaling Pathway

Ca^2+^ pathways are fundamental to the functioning of some mammalian peripheral clocks. One of them is in the skeletal muscle, in which clock genes are necessary for maintaining its phenotype and metabolic homeostasis (Lefta et al., 2011). In rodents, 3–16% of the skeletal muscle transcriptome exhibits a circadian oscillation and many of these genes code for proteins involved in Ca^2+^ signaling (McCarthy et al., 2007; Dyar et al., 2014). For instance, a microarray study evaluated the transcriptomic profile of hindlimb leg muscle in adult mice and observed rhythmic expression in Ca^2+^-calmodulin-dependent protein kinases (*camk*, *cask*), the Ca^2+^ transporter of the sarcoplasmic reticulum (*serca*), and the Ca^2+^ buffer protein, *parvalbumin*, which did not occur in the muscles of CLOCK-knockout mice (McCarthy et al., 2007). Similar results have recently been described for human primary myoblasts with reduced expression of CLOCK (Perrin et al., 2018). Interestingly, the hindlimb muscles of a muscle-specific BMAL1 knockout mouse continue to exhibit a significant proportion of cycling genes, and, when they are denervated, 15% of the genes then lose their rhythmic expression (Dyar et al., 2015). These findings suggest that extracellular signals –probably originating from the SCN–play a major role in imposing a daily oscillation to the skeletal muscle transcriptome. Importantly, the changes in gene expression that are dependent on innervation are mediated by Ca^2+^ signaling. Indeed, RNAseq data reveal that, in the mouse soleus, a slow muscle fiber, one of the circadian pathways that is enriched in response to nerve activity is the Ca^2+^-calcineurin-NFAT pathway (Dyar et al., 2015). Importantly, NFAT is a transcription factor with a key role in muscle adaptation in response to physical activity (Akimoto et al., 2005). NFAT translocates to the nucleus in a circadian manner, leading to the rhythmic expression of genes such as *rcan1*, a typical reporter of the Ca^2+^-calcineurin-NFAT axis, thus revealing that this signaling pathway plays a critical role in integrating external stimuli with cycling gene expression (Dyar et al., 2015).

The heart is another peripheral clock whose functioning is highly dependent on a rhythmic Ca^2+^ pathway. Indeed, Ca^2+^ homeostasis is fundamental to the functioning of the heart and alterations in Ca^2+^ signaling are associated with a variety of cardiac pathologies (Bers, 2014). In addition, the circadian clock is critical in order for myocytes to maintain their contractile and metabolic function. As a result, in rodents, for example, cardiac muscle cells exhibit rhythmic expression in many genes including those that code for proteins of the Ca^2+^ signaling pathway (Bray et al., 2008; Sachan et al., 2011). In particular, in the mouse, muscles of the left ventricle exhibit a daily rhythm in the translocation of NFAT into the nucleus and, as a result, also in RCAN1 expression (Sachan et al., 2011). Importantly, this effect is abolished by calcineurin inhibitors, indicating that the Ca^2+^/calcineurin/NFAT axis has rhythmic activity. Additionally, the functioning of SERCA is also under circadian control in mouse myocytes. In particular, phosphorylation of phospholamban (a SERCA inhibitory signaling protein) is restricted to the early part of the night, which prevents it from binding to SERCA (Sachan et al., 2011), thereby allowing the entry of Ca^2+^ into the endoplasmic reticulum in a circadian manner (Sachan et al., 2011).

Collectively, current evidence reveals that Ca^2+^ modulates the expression of clock genes and is in turn modulated directly or indirectly by the molecular clock machinery (Figure 2B). This relationship results in a rhythm of cytosolic Ca^2+^ levels and of its downstream effectors. As a result, Ca^2+^ signaling and core molecular clock components maintain a reciprocal relationship that is important for the proper functioning of biological clocks as well as for that of the underlying circadian regulation of physiology (see Graphical Abstract).

## Ca^2+^ Signaling and Clocks: Implications for Diseases and Chronomedicine

### Circadian Ca^2+^ Signaling and Diseases

In humans, circadian clocks impose a daily rhythm to many physiological processes and behaviors including sleep, blood hormone levels, locomotor activity, body core temperature, and metabolism (Levi and Schibler, 2007). In several neurological disorders, patients exhibit alterations in their circadian rhythms, which are associated with changes in the expression of core clock components. Such is the case for schizophrenia, depression, Parkinson’s disease (PD), and Alzheimer disease (AD), where patients display altered sleep, and melatonin and core body temperature rhythms, due to alterations in clock gene expression and the loss of synchronization among pacemaker cells in the SCN (Videnovic et al., 2014; Musiek and Holtzman, 2016). Similarly, a dysfunction in the circadian clock is linked with the pathogenesis of various types of human cancers (Sancar and van Gelder, 2021). What molecular mechanisms could link the disruption of circadian rhythms to the development of these diseases? As we discussed above, PKC and CaMK are integral components of the molecular clock and, remarkably, abnormalities in the activity of these proteins have been described in the early stages of disorders that are associated with disturbances in clock function. For instance, higher levels or abnormal activity of PKC and CaMK isozymes leads to neuronal cell death and a disruption in neuronal transmission in PD, AD, and in neuropsychiatric disorders (Mochly-Rosen et al., 2012; Robison, 2014). Similarly, PKC overexpression promotes angiogenesis and excessive cell proliferation in stomach, colon, and breast cancers (Mochly-Rosen et al., 2012). Thus, in these disorders, abnormal CaMK or PKC signaling may alter the functioning of circadian TTFLs of both central and peripherals clocks, leading to a disruption in the rhythm of a variety of behavioral and physiological processes. In fact, a recent review suggests that a dysfunction in the clock system may be causal of neurodegeneration and that the pharmacological regulation of TTFLs could improve clinical outcomes (Cederroth et al., 2019). Consistently, a variety of psychotropic and cancer chemopreventive agents act by modulating CaMK and PKC activity, respectively, (Celano et al., 2003; Mochly-Rosen et al., 2012). Thus, recovering the normal functioning of the circadian clocks could have important implications for the effectiveness of these treatments. Thus, future studies may focus on elucidating whether these Ca^2+^-associated proteins mediate the link between the disruption of circadian clocks and the pathogenesis of neurological disorders and cancer.

Ca^2+^ channels also play a role in neurological disorders that cause alterations in circadian rhythms. One of them is bipolar disorder (BD), which is characterized by mood instability and abnormalities in sleep and daily activity schedules (bedtime, waketime, and mealtime) (Alloy et al., 2017). In BD, lithium is the most effective mood stabilizer and corrects daily rhythms, but not all patients are responsive to this treatment (Alda, 2015). In particular, BD patients with longer circadian periodicities do not respond to lithium and exhibit a greater polypharmacy (Sanghani et al., 2020). Additionally, mice with a dysfunctional clock do not display typical lithium-induced behavioral changes. These findings suggest that in BD patients the mood stabilizing effects of lithium could be mediated by the circadian clock. Conversely, several variants of genes coding for proteins of the Ca^2+^ pathways including *cacna1c* (L-type VGCC) are risk alleles for BD (Gershon et al., 2014; Ament et al., 2015). Interestingly, fibroblasts from healthy humans, but not from BD patients, exhibit a rhythm in *cacna1c* expression, and the inactivation of this Ca^2+^ channel prevents lithium from increasing the amplitude of circadian rhythms (McCarthy et al., 2016). But VGCCs are not the only components of Ca^2+^ pathways that are relevant to the lithium-induced circadian response in BD patients. In particular, lithium lengthens the circadian period in cultured cells, which does not occur in the presence of an IP_3_R antagonist (McCarthy et al., 2019). In agreement with the potential role of IP_3_R, an allelic variant of this Ca^2+^ transporter potentiates the lithium-induced period lengthening in BD patient-derived fibroblasts, suggesting that a genetic factor in Ca^2+^ signaling could influence the circadian defects seen in BD patients (McCarthy et al., 2019). Given that the effects of lithium on the clock appear to correlate with its effectiveness in mood stabilization of BD patients (Sanghani et al., 2020), considering the allelic variants of Ca^2+^ signaling could be important for implementing personalized medicine that improves the effectiveness of BD treatments.

### Implications of Ca^2+^ Signaling in Chronomedicine

**FIGURE 3:**
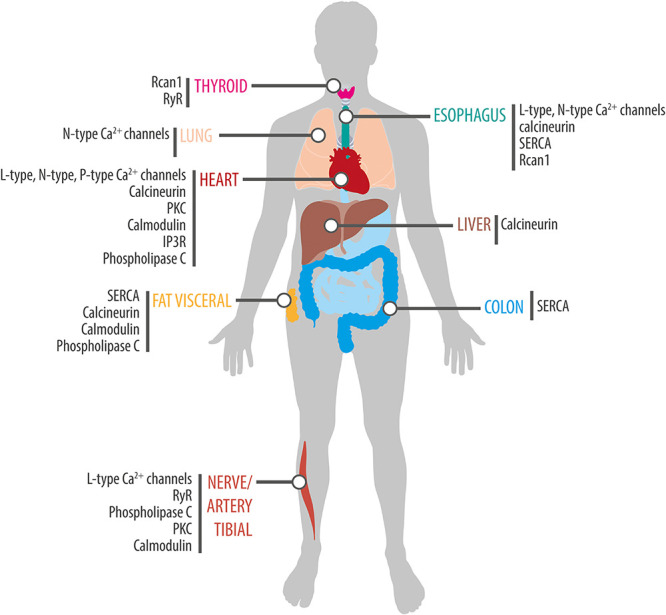
Rhythmic expression of components of the Ca^2+^ signaling pathway in human tissues. Schematic showing tissue-specific circadian expression of Ca^2+^-associated proteins and of Ca^2+^ channels and transporters in peripheral human tissues. Based on data from Ruben et al. (2018) (http://circadb.hogeneschlab.org/human).

In recent years, a growing number of studies have documented time of day effects in the effectiveness of medical interventions. Indeed, such variations have been reported for the success of treatments such as chemotherapy (Sancar and van Gelder, 2021), as well as in the efficacy of drugs used to treat several disease including hypertension, cancer, hypercholesterolemia, rheumatoid arthritis, allergies, sleep disturbances, and asthma (Cederroth et al., 2019). One explanation for this is that many of drugs have short half-lives and their targets exhibit circadian expression. For instance, in non-human primates, about 80% of protein-coding genes display a daily rhythm of expression and a large proportion of these genes are identified as drug targets by the United States Food and Drug Administration (Mure et al., 2018). Similarly, a genome-wide transcriptome study in humans identified thousands of genes with tissue-specific circadian expression in 13 different organs (Ruben et al., 2018). A notable example is the various L-type Ca^2+^ channel subunits, which display a rhythmic expression in the heart (Table 3 and Figure 3) suggesting that their sensitivity to drugs would vary during the course of the day. And indeed, nifedipine and verapamil, two Ca^2+^ channels blockers used as antihypertensive drugs, show an improved efficacy when administered before bedtime (White et al., 1995; Hermida et al., 2008). In addition, a variety of Ca^2+^ channels blockers have half-lives <6 h (Elliott and Ram, 2011) suggesting that taking these drugs at peak times of Ca^2+^ channel expression could improve the efficacy of the pharmacological therapy (this could also reduce side effects caused by actions on other tissues where the relevant targets cycle with a different phase). Consistent with this hypothesis, harmonizing the timing of administration of a drug with the time of peak expression of its target improves the effectiveness of treatments for cardiovascular disease, hypercholesterolemia, and obesity (Awad et al., 2017). On other hand, a circadian database generated by Ruben et al. (2018) shows that many genes encoding Ca^2+^ signaling proteins show daily rhythms of expression in a variety of peripheral clocks (Figure 3), which is consistent with previous transcriptomic analyses of different tissues and brain regions of mice (Zhang et al., 2014) and baboons (Mure et al., 2018). Thus, taking into account the daily changes in the abundance of these drug targets may have significant implications for medicine because proteins from the Ca^2+^ pathway are therapeutic targets in multiple disorders. For instance, the SERCA inhibitor prodrug, mipsagargin, is being assessed in clinical trials for the treatment of various types of cancers including prostate and liver (Mahalingam et al., 2016; Mahalingam et al., 2019). Similarly, recent pre-clinical studies propose that the RyR antagonist, dantrolene, would have a neuroprotective role in Huntington and Alzheimer’s models (Chen et al., 2011; Liang and Wei, 2015).

**TABLE 3:** Drugs targeting elements of the Ca^2+^ signaling pathway and their usage in medicine.

**Therapeutic target**	**Tissue-specific circadian expression^a^**	**Acrophase^b^**	**Drug**	**Clinical use**
L-type Ca^2+^ channel subunits	Heart, artery tibial, nerve tibial, subcutaneous fat (SF)	Morning (nerve tibial, SF), night (heart and nerve tibial)	Verapamil, nifedipine,	Antihypertensive, cardiac arrhythmia, angina (Striessnig et al., 2015)
N-type Ca^2+^ channel subunits	Heart, esophagus, lung	Night (heart and lung) and morning (esophagus)	Ziconotide, nicardipine	Analgesic (McGivern, 2007), antihypertensive (Elliott and Ram, 2011)
P-type Ca^2+^ channel subunits	Heart, pituitary	Night (pituitary and heart)	Verapamil, flunarizine, levetiracetam	Antihypertensive (Elliott and Ram, 2011), epilepsy (Hasan et al., 2013)
calcineurin	Esophagus, liver	Morning (esophagus and liver)	Cyclosporin	Immunosuppression, rheumatoid arthritis (Tedesco and Haragsim, 2012)
Serca	Esophagus, colon, fat visceral	Morning (esophagus), evening (colon and fat visceral)	Mipsagargin	Cancer (clinical trials) (Mahalingam et al., 2016)
Ryr	Thyroid, nerve tibial	Morning (nerve tibial), night (thyroid)	Dantrolene	Neuroleptic malignant syndrome (Krause et al., 2004)
PKC	Heart, artery tibial, skin	morning (artery tibial, skin), evening (heart)	Tamoxifen	Cancer (Mochly-Rosen et al., 2012)
Rcan1	Thyroid, esophagus	Morning (thyroid and esophagus)	Not described	Not described
Calmodulin	Artery tibial, fat visceral	Morning (artery tibial and fat visceral)	Loperamide, fluvoxamine	Shizophrenia (Celano et al., 2003), diarrhea (Hanauer, 2008)
IP3R	Heart, lung	Morning (heart), night (lung)	Not described	Not described
Phospholipase C	Heart, nerve tibial, fat visceral	Morning (fat visceral and nerve tibial), night (heart)	Not described	Not described

*^a^Circadian expression according to database generated by RNAseq and cyclic ordering by periodic structure (CYCLOPS) (Ruben et al., 2018) (http://circadb.hogeneschlab.org/human).*

*^b^Acrophase of expression for each gene was based on criteria used in CYCLOPS. As reference, 0 radians is close of the time of peak of expression of BMAL (Zeitgeber time 10) (Ron Anafli, personal communication).*

Considering the daily changes in Ca^2+^ signaling may be relevant not only for deciding on the best time for drug administration but also for scheduling medical interventions. For example, in humans, a common outcome of cardiac surgery is myocardial injury due to ischemia-reperfusion (I/R) (Hausenloy and Yellon, 2016). A recent study in humans, revealed that the injury by I/R following aortic valve replacement is greatest when the surgery is done in the morning compared to the evening. This difference in the time of the day is abolished in a mouse model of hypoxia/reoxygenation treated with Rev-ERBα antagonists, suggesting a critical role of this circadian clock component in the myocardial tolerance to I/R (Montaigne et al., 2018). Interestingly, this circadian regulation would not only be controlled by Rev-ERBα but also by the calcineurin/rcan1 axis. As we discussed above, in mice, the heart calcineurin/NFAT/Rcan1 pathway displays rhythmic activity (Sachan et al., 2011) and, remarkably, this process could influence the daily variations in the myocardial tolerance to I/R. Unlike the findings reported by Montaigne et al. (2018), injury by I/R following coronary artery surgery in mice is greater in animals operated at the end of their active phase compared to those operated at the beginning of this phase (Rotter et al., 2014). This time of day difference in tolerance to I/R is not present in Rcan1 knockout mice (Rotter et al., 2014). Since these mutant mice display a rhythmic expression in canonical clock genes in the heart, these results suggest that Rcan1 mediates this effect without altering the global functioning of this peripheral clock. In addition, inhibiting calcineurin activity decreases the severity of the I/R injury in animals operated at PM whereas it does not confer additional protection in AM-operated mice, supporting the idea that the calcineurin/Rcan1 pathway plays a key role in determining the time of day-dependent susceptibility of the heart to I/R. Even though additional studies are needed to elucidate whether a similar regulation occurs in humans, this finding positions the calcineurin/rcan1 axis as a therapeutic target for cardioprotection and for blunting the effect of the time of day on the outcome of cardiac surgery.

Collectively these findings provide strong evidence that the circadian control of Ca^2+^ signaling in biological clocks may have significant implications for medicine (Table 3). Thus, taking the 24-h dynamics of Ca^2+^ signaling into account in pharmacological therapies and medical interventions could improve drug efficacy and postoperative clinical outcomes, supporting the emerging idea that circadian biology should be an integral part of translational research (Cederroth et al., 2019).

## Closing Remarks

We currently have a very detailed understanding of how circadian clocks function. By comparison, much less is known about how they are synchronized and coordinated; how they transmit time information to the host; and, in turn, how they can be affected by the host’s physiology and behavior. All of these system-level functions ultimately converge on intracellular signals that affect gene expression, which, in turn, alter the activity of clocks, and the physiology of cells, organs, and organisms. Future work aimed at increasing our knowledge on how intracellular signaling regulates, and is regulated, by clocks will be important for furthering our understanding of how this intricate network of interactions is effected and how a unified time for the organism is maintained yet can be modulated by the host. It will also be critical for the development of more effective chronomedicine prescriptions aimed at improving the effectiveness of drugs while reducing their negative and off-target effects.

## Author Contributions

JC-L wrote the first draft. JC-L and JE edited manuscript. Both authors contributed to the article and approved the submitted version.

## Conflict of Interest

The authors declare that the research was conducted in the absence of any commercial or financial relationships that could be construed as a potential conflict of interest.

## References

[B1] Abruzzi K. C. Zadina A. Luo W. Wiyanto E. Rahman R. Guo F. (2017). RNA-seq analysis of *Drosophila* clock and non-clock neurons reveals neuron-specific cycling and novel candidate neuropeptides. *PLoS Genet.* 13:e1006613. 10.1371/journal.pgen.1006613 28182648 PMC5325595

[B2] Agostino P. V. Ferreyra G. A. Murad A. D. Watanabe Y. Golombek D. A. (2004). Diurnal, circadian and photic regulation of calcium/calmodulin-dependent kinase II and neuronal nitric oxide synthase in the hamster suprachiasmatic nuclei. *Neurochem. Int.* 44 617–625. 10.1016/j.neuint.2003.09.005 15016477

[B3] Aguilar-Roblero R. Mercado C. Alamilla J. Laville A. Diaz-Munoz M. (2007). Ryanodine receptor Ca2+-release channels are an output pathway for the circadian clock in the rat suprachiasmatic nuclei. *Eur. J. Neurosci.* 26 575–582. 10.1111/j.1460-9568.2007.05679.x 17686038

[B4] Akashi M. Tsuchiya Y. Yoshino T. Nishida E. (2002). Control of intracellular dynamics of mammalian period proteins by casein kinase I epsilon (CKIepsilon) and CKIdelta in cultured cells. *Mol. Cell. Biol.* 22 1693–1703. 10.1128/mcb.22.6.1693-1703.2002 11865049 PMC135601

[B5] Akimoto T. Pohnert S. C. Li P. Zhang M. Gumbs C. Rosenberg P. B. (2005). Exercise stimulates Pgc-1alpha transcription in skeletal muscle through activation of the p38 MAPK pathway. *J. Biol. Chem.* 280 19587–19593. 10.1074/jbc.m408862200 15767263

[B6] Alda M. (2015). Lithium in the treatment of bipolar disorder: pharmacology and pharmacogenetics. *Mol. Psychiatry* 20 661–670. 10.1038/mp.2015.4 25687772 PMC5125816

[B7] Alloy L. B. Ng T. H. Titone M. K. Boland E. M. (2017). Circadian rhythm dysregulation in bipolar spectrum disorders. *Curr. Psychiatry Rep.* 19:21. 10.1007/s11920-017-0772-z PMC6661150 28321642

[B8] Ament S. A. Szelinger S. Glusman G. Ashworth J. Hou L. Akula N. (2015). Rare variants in neuronal excitability genes influence risk for bipolar disorder. *Proc. Natl. Acad. Sci. U.S.A.* 112 3576–3581. 10.1073/pnas.1424958112 25730879 PMC4371952

[B9] Ampleford E. J. Steel C. G. (1985). Circadian control of a daily rhythm in hemolymph ecdysteroid titer in the insect *Rhodnius prolixus* (Hemiptera). *Gen. Comp. Endocrinol.* 59 453–459. 10.1016/0016-6480(85)90404-6 4043725

[B10] Awad K. Serban M. C. Penson P. Mikhailidis D. P. Toth P. P. Jones S. R. (2017). Effects of morning vs evening statin administration on lipid profile: a systematic review and meta-analysis. *J. Clin. Lipidol.* 11 972–985.e9. 28826569 10.1016/j.jacl.2017.06.001

[B11] Baez-Ruiz A. Diaz-Munoz M. (2011). Chronic inhibition of endoplasmic reticulum calcium-release channels and calcium-ATPase lengthens the period of hepatic clock gene Per1. *J. Circadian Rhythms* 9:6. 10.1186/1740-3391-9-6 21740569 PMC3142245

[B12] Bers D. M. (2014). Cardiac sarcoplasmic reticulum calcium leak: basis and roles in cardiac dysfunction. *Annu. Rev. Physiol.* 76 107–127. 10.1146/annurev-physiol-020911-153308 24245942

[B13] Brancaccio M. Maywood E. S. Chesham J. E. Loudon A. S. Hastings M. H. (2013). A Gq-Ca2+ axis controls circuit-level encoding of circadian time in the suprachiasmatic nucleus. *Neuron* 78 714–728. 10.1016/j.neuron.2013.03.011 23623697 PMC3666084

[B14] Brancaccio M. Patton A. P. Chesham J. E. Maywood E. S. Hastings M. H. (2017). Astrocytes control circadian timekeeping in the suprachiasmatic nucleus via glutamatergic signaling. *Neuron* 93 1420–1435.e5. 28285822 10.1016/j.neuron.2017.02.030 PMC5376383

[B15] Bray M. S. Shaw C. A. Moore M. W. Garcia R. A. Zanquetta M. M. Durgan D. J. (2008). Disruption of the circadian clock within the cardiomyocyte influences myocardial contractile function, metabolism, and gene expression. *Am. J. Physiol. Heart Circ. Physiol.* 294 H1036–H1047. 18156197 10.1152/ajpheart.01291.2007

[B16] Cederroth C. R. Albrecht U. Bass J. Brown S. A. Dyhrfjeld-Johnsen J. Gachon F. (2019). Medicine in the fourth dimension. *Cell Metab.* 30 238–250. 31390550 10.1016/j.cmet.2019.06.019 PMC6881776

[B17] Celano E. Tiraboschi E. Consogno E. D’urso G. Mbakop M. P. Gennarelli M. (2003). Selective regulation of presynaptic calcium/calmodulin-dependent protein kinase II by psychotropic drugs. *Biol. Psychiatry* 53 442–449. 10.1016/s0006-3223(02)01491-9 12614997

[B18] Chen X. Wu J. Lvovskaya S. Herndon E. Supnet C. Bezprozvanny I. (2011). Dantrolene is neuroprotective in Huntington’s disease transgenic mouse model. *Mol. Neurodegener.* 6:81. 10.1186/1750-1326-6-81 22118545 PMC3235068

[B19] Cheng H. Y. Papp J. W. Varlamova O. Dziema H. Russell B. Curfman J. P. (2007). microRNA modulation of circadian-clock period and entrainment. *Neuron* 54 813–829. 10.1016/j.neuron.2007.05.017 17553428 PMC2590749

[B20] Colwell C. S. (2000). Circadian modulation of calcium levels in cells in the suprachiasmatic nucleus. *Eur. J. Neurosci.* 12 571–576. 10.1046/j.1460-9568.2000.00939.x 10712636 PMC4353598

[B21] Colwell C. S. (2011). Linking neural activity and molecular oscillations in the SCN. *Nat. Rev. Neurosci.* 12 553–569. 10.1038/nrn3086 21886186 PMC4356239

[B22] Colwell C. S. Whitmore D. Michel S. Block G. D. (1994). Calcium plays a central role in phase shifting the ocular circadian pacemaker of *Aplysia*. *J. Comp. Physiol. A* 175 415–423. 7965916 10.1007/BF00199249

[B23] Damiola F. Le Minh N. Preitner N. Kornmann B. Fleury-Olela F. Schibler U. (2000). Restricted feeding uncouples circadian oscillators in peripheral tissues from the central pacemaker in the suprachiasmatic nucleus. *Genes Dev.* 14 2950–2961. 10.1101/gad.183500 11114885 PMC317100

[B24] Depetris-Chauvin A. Berni J. Aranovich E. J. Muraro N. I. Beckwith E. J. Ceriani M. F. (2011). Adult-specific electrical silencing of pacemaker neurons uncouples molecular clock from circadian outputs. *Curr. Biol.* 21 1783–1793. 10.1016/j.cub.2011.09.027 22018542 PMC3226771

[B25] Ding J. M. Buchanan G. F. Tischkau S. A. Chen D. Kuriashkina L. Faiman L. E. (1998). A neuronal ryanodine receptor mediates light-induced phase delays of the circadian clock. *Nature* 394 381–384. 10.1038/28639 9690474

[B26] Dyar K. A. Ciciliot S. Tagliazucchi G. M. Pallafacchina G. Tothova J. Argentini C. (2015). The calcineurin-NFAT pathway controls activity-dependent circadian gene expression in slow skeletal muscle. *Mol. Metab.* 4 823–833. 10.1016/j.molmet.2015.09.004 26629406 PMC4632177

[B27] Dyar K. A. Ciciliot S. Wright L. E. Bienso R. S. Tagliazucchi G. M. Patel V. R. (2014). Muscle insulin sensitivity and glucose metabolism are controlled by the intrinsic muscle clock. *Mol. Metab.* 3 29–41. 10.1016/j.molmet.2013.10.005 24567902 PMC3929910

[B28] Eide E. J. Woolf M. F. Kang H. Woolf P. Hurst W. Camacho F. (2005). Control of mammalian circadian rhythm by CKIepsilon-regulated proteasome-mediated PER2 degradation. *Mol. Cell. Biol.* 25 2795–2807. 10.1128/mcb.25.7.2795-2807.2005 15767683 PMC1061645

[B29] Elliott W. J. Ram C. V. (2011). Calcium channel blockers. *J. Clin. Hypertens. (Greenwich)* 13 687–689. 10.1111/j.1751-7176.2011.00513.x 21896151 PMC8108866

[B30] Enoki R. Kuroda S. Ono D. Hasan M. T. Ueda T. Honma S. (2012). Topological specificity and hierarchical network of the circadian calcium rhythm in the suprachiasmatic nucleus. *Proc. Natl. Acad. Sci. U.S.A.* 109 21498–21503. 10.1073/pnas.1214415110 23213253 PMC3535646

[B31] Enoki R. Ono D. Kuroda S. Honma S. Honma K. I. (2017). Dual origins of the intracellular circadian calcium rhythm in the suprachiasmatic nucleus. *Sci. Rep.* 7:41733. 10.1038/srep41733 PMC5290527 28155916

[B32] Gallego M. Virshup D. M. (2007). Post-translational modifications regulate the ticking of the circadian clock. *Nat. Rev. Mol. Cell Biol.* 8 139–148. 10.1038/nrm2106 17245414

[B33] Gershon E. S. Grennan K. Busnello J. Badner J. A. Ovsiew F. Memon S. (2014). A rare mutation of CACNA1C in a patient with bipolar disorder, and decreased gene expression associated with a bipolar-associated common SNP of CACNA1C in brain. *Mol. Psychiatry* 19 890–894. 10.1038/mp.2013.107 23979604 PMC4151967

[B34] Ginty D. D. Kornhauser J. M. Thompson M. A. Bading H. Mayo K. E. Takahashi J. S. (1993). Regulation of CREB phosphorylation in the suprachiasmatic nucleus by light and a circadian clock. *Science* 260 238–241. 10.1126/science.8097062 8097062

[B35] Guo F. Chen X. Rosbash M. (2017). Temporal calcium profiling of specific circadian neurons in freely moving flies. *Proc. Natl. Acad. Sci. U.S.A.* 114 E8780–E8787. 28973886 10.1073/pnas.1706608114 PMC5642695

[B36] Hablitz L. M. Gunesch A. N. Cravetchi O. Moldavan M. Allen C. N. (2020). Cannabinoid signaling recruits astrocytes to modulate presynaptic function in the suprachiasmatic nucleus. *eNeuro* 7:ENEURO.0081-19.2020. 10.1523/ENEURO.0081-19.2020 PMC7029187 31964686

[B37] Hanauer S. B. (2008). The role of loperamide in gastrointestinal disorders. *Rev. Gastroenterol. Disord.* 8 15–20. 18477966

[B38] Hao H. Allen D. L. Hardin P. E. (1997). A circadian enhancer mediates PER-dependent mRNA cycling in *Drosophila melanogaster*. *Mol. Cell. Biol.* 17 3687–3693. 10.1128/mcb.17.7.3687 9199302 PMC232220

[B39] Hardin P. E. (2011). Molecular genetic analysis of circadian timekeeping in *Drosophila*. *Adv. Genet.* 74 141–173. 10.1016/b978-0-12-387690-4.00005-2 21924977 PMC4108082

[B40] Harrisingh M. C. Wu Y. Lnenicka G. A. Nitabach M. N. (2007). Intracellular Ca2+ regulates free-running circadian clock oscillation in vivo. *J. Neurosci.* 27 12489–12499. 10.1523/jneurosci.3680-07.2007 18003827 PMC6673328

[B41] Hasan M. Pulman J. Marson A. G. (2013). Calcium antagonists as an add-on therapy for drug-resistant epilepsy. *Cochrane Database Syst. Rev.* 2013:CD002750. 10.1002/14651858.CD002750.pub2 PMC7100543 23543516

[B42] Hausenloy D. J. Yellon D. M. (2016). Ischaemic conditioning and reperfusion injury. *Nat. Rev. Cardiol.* 13 193–209. 10.1038/nrcardio.2016.5 26843289

[B43] Helfrich-Forster C. Shafer O. T. Wulbeck C. Grieshaber E. Rieger D. Taghert P. (2007). Development and morphology of the clock-gene-expressing lateral neurons of *Drosophila melanogaster*. *J. Comp. Neurol.* 500 47–70. 10.1002/cne.21146 17099895

[B44] Hermida R. C. Ayala D. E. Mojon A. Fernandez J. R. (2008). Chronotherapy with nifedipine GITS in hypertensive patients: improved efficacy and safety with bedtime dosing. *Am. J. Hypertens.* 21 948–954. 10.1038/ajh.2008.216 18600215

[B45] Huang X. Warren J. T. Gilbert L. I. (2008). New players in the regulation of ecdysone biosynthesis. *J. Genet. Genomics* 35 1–10. 10.1016/s1673-8527(08)60001-6 18222403

[B46] Hyun S. Lee Y. Hong S. T. Bang S. Paik D. Kang J. (2005). *Drosophila* GPCR han is a receptor for the circadian clock neuropeptide PDF. *Neuron* 48 267–278. 10.1016/j.neuron.2005.08.025 16242407

[B47] Ikeda M. Ikeda M. (2014). Bmal1 is an essential regulator for circadian cytosolic Ca(2)(+) rhythms in suprachiasmatic nucleus neurons. *J. Neurosci.* 34 12029–12038. 10.1523/jneurosci.5158-13.2014 25186748 PMC6608459

[B48] Ikeda M. Sugiyama T. Wallace C. S. Gompf H. S. Yoshioka T. Miyawaki A. (2003). Circadian dynamics of cytosolic and nuclear Ca2+ in single suprachiasmatic nucleus neurons. *Neuron* 38 253–263. 10.1016/s0896-6273(03)00164-8 12718859

[B49] Jakubcakova V. Oster H. Tamanini F. Cadenas C. Leitges M. Van Der Horst G. T. (2007). Light entrainment of the mammalian circadian clock by a PRKCA-dependent posttranslational mechanism. *Neuron* 54 831–843. 10.1016/j.neuron.2007.04.031 17553429

[B50] Jones J. R. Simon T. Lones L. Herzog E. D. (2018). SCN VIP neurons are essential for normal light-mediated resetting of the circadian system. *J. Neurosci.* 38 7986–7995. 10.1523/jneurosci.1322-18.2018 30082421 PMC6596148

[B51] King A. N. Sehgal A. (2020). Molecular and circuit mechanisms mediating circadian clock output in the *Drosophila* brain. *Eur. J. Neurosci.* 51 268–281. 10.1111/ejn.14092 30059181 PMC6353709

[B52] Kon N. Yoshikawa T. Honma S. Yamagata Y. Yoshitane H. Shimizu K. (2014). CaMKII is essential for the cellular clock and coupling between morning and evening behavioral rhythms. *Genes Dev.* 28 1101–1110. 10.1101/gad.237511.114 24831701 PMC4035538

[B53] Kozlov A. Nagoshi E. (2019). Decoding *Drosophila* circadian pacemaker circuit. *Curr. Opin. Insect Sci.* 36 33–38. 10.1016/j.cois.2019.06.010 31376574

[B54] Krause T. Gerbershagen M. U. Fiege M. Weisshorn R. Wappler F. (2004). Dantrolene–a review of its pharmacology, therapeutic use and new developments. *Anaesthesia* 59 364–373. 10.1111/j.1365-2044.2004.03658.x 15023108

[B55] Kweon S. H. Lee J. Lim C. Choe J. (2018). High-amplitude circadian rhythms in *Drosophila* driven by calcineurin-mediated post-translational control of sarah. *Genetics* 209 815–828. 10.1534/genetics.118.300808 29724861 PMC6028259

[B56] Lefta M. Wolff G. Esser K. A. (2011). Circadian rhythms, the molecular clock, and skeletal muscle. *Curr. Top. Dev. Biol.* 96 231–271. 10.1016/b978-0-12-385940-2.00009-7 21621073 PMC4545213

[B57] Levi F. Schibler U. (2007). Circadian rhythms: mechanisms and therapeutic implications. *Annu. Rev. Pharmacol. Toxicol.* 47 593–628. 10.1146/annurev.pharmtox.47.120505.105208 17209800

[B58] Liang L. Wei H. (2015). Dantrolene, a treatment for Alzheimer disease? *Alzheimer Dis. Assoc. Disord.* 29 1–5. 10.1097/wad.0000000000000076 25551862 PMC4334699

[B59] Liang X. Holy T. E. Taghert P. H. (2016). Synchronous *Drosophila* circadian pacemakers display nonsynchronous Ca(2)(+) rhythms in vivo. *Science* 351 976–981. 10.1126/science.aad3997 26917772 PMC4836443

[B60] Liang X. Holy T. E. Taghert P. H. (2017). A series of suppressive signals within the *Drosophila* circadian neural circuit generates sequential daily outputs. *Neuron* 94 1173–1189.e4. 28552314 10.1016/j.neuron.2017.05.007 PMC5502710

[B61] Lundkvist G. B. Kwak Y. Davis E. K. Tei H. Block G. D. (2005). A calcium flux is required for circadian rhythm generation in mammalian pacemaker neurons. *J. Neurosci.* 25 7682–7686. 10.1523/jneurosci.2211-05.2005 16107654 PMC6725395

[B62] Mahalingam D. Peguero J. Cen P. Arora S. P. Sarantopoulos J. Rowe J. (2019). A phase II, multicenter, single-arm study of mipsagargin (G-202) as a second-line therapy following sorafenib for adult patients with progressive advanced hepatocellular carcinoma. *Cancers (Basel)* 11:833. 10.3390/cancers11060833 31212948 PMC6627768

[B63] Mahalingam D. Wilding G. Denmeade S. Sarantopoulas J. Cosgrove D. Cetnar J. (2016). Mipsagargin, a novel thapsigargin-based PSMA-activated prodrug: results of a first-in-man phase I clinical trial in patients with refractory, advanced or metastatic solid tumours. *Br. J. Cancer* 114 986–994. 10.1038/bjc.2016.72 27115568 PMC4984914

[B64] McCarthy J. J. Andrews J. L. Mcdearmon E. L. Campbell K. S. Barber B. K. Miller B. H. (2007). Identification of the circadian transcriptome in adult mouse skeletal muscle. *Physiol. Genomics* 31 86–95. 10.1152/physiolgenomics.00066.2007 17550994 PMC6080860

[B65] McCarthy M. J. Le Roux M. J. Wei H. Beesley S. Kelsoe J. R. Welsh D. K. (2016). Calcium channel genes associated with bipolar disorder modulate lithium’s amplification of circadian rhythms. *Neuropharmacology* 101 439–448. 10.1016/j.neuropharm.2015.10.017 26476274 PMC4681663

[B66] McCarthy M. J. Wei H. Nievergelt C. M. Stautland A. Maihofer A. X. Welsh D. K. (2019). Chronotype and cellular circadian rhythms predict the clinical response to lithium maintenance treatment in patients with bipolar disorder. *Neuropsychopharmacology* 44 620–628. 30487653 10.1038/s41386-018-0273-8 PMC6333516

[B67] McGivern J. G. (2007). Ziconotide: a review of its pharmacology and use in the treatment of pain. *Neuropsychiatr. Dis. Treat.* 3 69–85. 10.2147/nedt.2007.3.1.69 19300539 PMC2654521

[B68] Mizrak D. Ruben M. Myers G. N. Rhrissorrakrai K. Gunsalus K. C. Blau J. (2012). Electrical activity can impose time of day on the circadian transcriptome of pacemaker neurons. *Curr. Biol.* 22 1871–1880. 10.1016/j.cub.2012.07.070 22940468 PMC3562355

[B69] Mochly-Rosen D. Das K. Grimes K. V. (2012). Protein kinase C, an elusive therapeutic target? *Nat. Rev. Drug Discov.* 11 937–957. 10.1038/nrd3871 23197040 PMC3760692

[B70] Mohawk J. A. Green C. B. Takahashi J. S. (2012). Central and peripheral circadian clocks in mammals. *Annu. Rev. Neurosci.* 35 445–462. 10.1146/annurev-neuro-060909-153128 22483041 PMC3710582

[B71] Montaigne D. Marechal X. Modine T. Coisne A. Mouton S. Fayad G. (2018). Daytime variation of perioperative myocardial injury in cardiac surgery and its prevention by Rev-Erbalpha antagonism: a single-centre propensity-matched cohort study and a randomised study. *Lancet* 391 59–69. 10.1016/s0140-6736(17)32132-3 29107324

[B72] Morioka E. Matsumoto A. Ikeda M. (2012). Neuronal influence on peripheral circadian oscillators in pupal *Drosophila* prothoracic glands. *Nat. Commun.* 3:909. 10.1038/ncomms1922 PMC3621432 22713751

[B73] Mure L. S. Le H. D. Benegiamo G. Chang M. W. Rios L. Jillani N. (2018). Diurnal transcriptome atlas of a primate across major neural and peripheral tissues. *Science* 359:eaao0318. 10.1126/science.aao0318 29439024 PMC5924732

[B74] Musiek E. S. Holtzman D. M. (2016). Mechanisms linking circadian clocks, sleep, and neurodegeneration. *Science* 354 1004–1008. 10.1126/science.aah4968 27885006 PMC5219881

[B75] Nahm S. S. Farnell Y. Z. Griffith W. Earnest D. J. (2005). Circadian regulation and function of voltage-dependent calcium channels in the suprachiasmatic nucleus. *J. Neurosci.* 25 9304–9308. 10.1523/jneurosci.2733-05.2005 16207890 PMC6725766

[B76] Nam H. J. Boo K. Kim D. Han D. H. Choe H. K. Kim C. R. (2014). Phosphorylation of LSD1 by PKCalpha is crucial for circadian rhythmicity and phase resetting. *Mol. Cell* 53 791–805. 10.1016/j.molcel.2014.01.028 24582500

[B77] Noguchi T. Leise T. L. Kingsbury N. J. Diemer T. Wang L. L. Henson M. A. (2017). Calcium circadian rhythmicity in the suprachiasmatic nucleus: cell autonomy and network modulation. *eNeuro* 4:ENEURO.0160-17.2017. 10.1523/ENEURO.0160-17.2017 PMC5562299 28828400

[B78] Nomura K. Takeuchi Y. Yamaguchi S. Okamura H. Fukunaga K. (2003). Involvement of calcium/calmodulin-dependent protein kinase II in the induction of mPer1. *J. Neurosci. Res.* 72 384–392. 10.1002/jnr.10581 12692905

[B79] Ogueta M. Hardie R. C. Stanewsky R. (2018). Non-canonical phototransduction mediates synchronization of the *Drosophila melanogaster* circadian clock and retinal light responses. *Curr. Biol.* 28 1725–1735.e3. 29779871 10.1016/j.cub.2018.04.016 PMC5988559

[B80] Ogueta M. Hardie R. C. Stanewsky R. (2020). Light sampling via throttled visual phototransduction robustly synchronizes the *Drosophila* circadian clock. *Curr. Biol.* 30 2551–2563.e3. 32502413 10.1016/j.cub.2020.04.067

[B81] Palacios-Munoz A. Ewer J. (2018). Calcium and cAMP directly modulate the speed of the *Drosophila* circadian clock. *PLoS Genet.* 14:e1007433. 10.1371/journal.pgen.1007433 29879123 PMC6007936

[B82] Panda S. Antoch M. P. Miller B. H. Su A. I. Schook A. B. Straume M. (2002). Coordinated transcription of key pathways in the mouse by the circadian clock. *Cell* 109 307–320. 10.1016/s0092-8674(02)00722-5 12015981

[B83] Patke A. Young M. W. Axelrod S. (2020). Molecular mechanisms and physiological importance of circadian rhythms. *Nat. Rev. Mol. Cell Biol.* 21 67–84. 10.1038/s41580-019-0179-2 31768006

[B84] Pembroke W. G. Babbs A. Davies K. E. Ponting C. P. Oliver P. L. (2015). Temporal transcriptomics suggest that twin-peaking genes reset the clock. *Elife* 4:e10518. 10.7554/eLife.10518 PMC4718813 26523393

[B85] Perrin L. Loizides-Mangold U. Chanon S. Gobet C. Hulo N. Isenegger L. (2018). Transcriptomic analyses reveal rhythmic and CLOCK-driven pathways in human skeletal muscle. *Elife* 7:e34114. 10.7554/eLife.34114 PMC5902165 29658882

[B86] Pfeffer M. Muller C. M. Mordel J. Meissl H. Ansari N. Deller T. (2009). The mammalian molecular clockwork controls rhythmic expression of its own input pathway components. *J. Neurosci.* 29 6114–6123. 10.1523/jneurosci.0275-09.2009 19439589 PMC6665491

[B87] Preitner N. Damiola F. Lopez-Molina L. Zakany J. Duboule D. Albrecht U. (2002). The orphan nuclear receptor REV-ERBalpha controls circadian transcription within the positive limb of the mammalian circadian oscillator. *Cell* 110 251–260. 10.1016/s0092-8674(02)00825-5 12150932

[B88] Reischl S. Kramer A. (2011). Kinases and phosphatases in the mammalian circadian clock. *FEBS Lett.* 585 1393–1399. 10.1016/j.febslet.2011.02.038 21376720

[B89] Rivera-Bermudez M. A. Gerdin M. J. Earnest D. J. Dubocovich M. L. (2003). Regulation of basal rhythmicity in protein kinase C activity by melatonin in immortalized rat suprachiasmatic nucleus cells. *Neurosci. Lett.* 346 37–40. 10.1016/s0304-3940(03)00590-1 12850542

[B90] Robison A. J. (2014). Emerging role of CaMKII in neuropsychiatric disease. *Trends Neurosci.* 37 653–662. 10.1016/j.tins.2014.07.001 25087161

[B91] Robles M. S. Boyault C. Knutti D. Padmanabhan K. Weitz C. J. (2010). Identification of RACK1 and protein kinase Calpha as integral components of the mammalian circadian clock. *Science* 327 463–466. 10.1126/science.1180067 20093473

[B92] Rotter D. Grinsfelder D. B. Parra V. Pedrozo Z. Singh S. Sachan N. (2014). Calcineurin and its regulator, RCAN1, confer time-of-day changes in susceptibility of the heart to ischemia/reperfusion. *J. Mol. Cell. Cardiol.* 74 103–111. 10.1016/j.yjmcc.2014.05.004 24838101 PMC4114994

[B93] Ruben M. D. Wu G. Smith D. F. Schmidt R. E. Francey L. J. Lee Y. Y. (2018). A database of tissue-specific rhythmically expressed human genes has potential applications in circadian medicine. *Sci. Transl. Med.* 10:eaat8806. 10.1126/scitranslmed.aat8806 30209245 PMC8961342

[B94] Sachan N. Dey A. Rotter D. Grinsfelder D. B. Battiprolu P. K. Sikder D. (2011). Sustained hemodynamic stress disrupts normal circadian rhythms in calcineurin-dependent signaling and protein phosphorylation in the heart. *Circ. Res.* 108 437–445. 10.1161/circresaha.110.235309 21233454 PMC3042501

[B95] Saint-Charles A. Michard-Vanhee C. Alejevski F. Chelot E. Boivin A. Rouyer F. (2016). Four of the six *Drosophila* rhodopsin-expressing photoreceptors can mediate circadian entrainment in low light. *J. Comp. Neurol.* 524 2828–2844. 10.1002/cne.23994 26972685

[B96] Sancar A. van Gelder R. N. (2021). Clocks, cancer, and chronochemotherapy. *Science* 371:eabb0738. 10.1126/science.abb0738 33384351

[B97] Sanghani H. R. Jagannath A. Humberstone T. Ebrahimjee F. Thomas J. M. Churchill G. C. (2020). Patient fibroblast circadian rhythms predict lithium sensitivity in bipolar disorder. *Mol. Psychiatry* 10.1038/s41380-020-0769-6 32404948 PMC8589670

[B98] Schibler U. Gotic I. Saini C. Gos P. Curie T. Emmenegger Y. (2015). Clock-talk: interactions between central and peripheral circadian oscillators in mammals. *Cold Spring Harb. Symp. Quant. Biol.* 80 223–232. 10.1101/sqb.2015.80.027490 26683231

[B99] Schmutz I. Chavan R. Ripperger J. A. Maywood E. S. Langwieser N. Jurik A. (2014). A specific role for the REV-ERBalpha-controlled L-type voltage-gated calcium channel CaV1.2 in resetting the circadian clock in the late night. *J. Biol. Rhythms* 29 288–298. 10.1177/0748730414540453 25238857 PMC4608047

[B100] Selcho M. Millan C. Palacios-Munoz A. Ruf F. Ubillo L. Chen J. (2017). Central and peripheral clocks are coupled by a neuropeptide pathway in *Drosophila*. *Nat. Commun.* 8:15563. 10.1038/ncomms15563 PMC5459987 28555616

[B101] Shi L. Ko M. L. Ko G. Y. (2009). Rhythmic expression of microRNA-26a regulates the L-type voltage-gated calcium channel alpha1C subunit in chicken cone photoreceptors. *J. Biol. Chem.* 284 25791–25803. 10.1074/jbc.m109.033993 19608742 PMC2757981

[B102] Smith W. Rybczynski R. Gilbert L. (2012). *Insect Endocrinology.* New York, NY: Academic Press.

[B103] Stadler F. Schmutz I. Schwaller B. Albrecht U. (2010). Lack of calbindin-D28k alters response of the murine circadian clock to light. *Chronobiol. Int.* 27 68–82. 10.3109/07420521003648554 20205558

[B104] Striessnig J. Ortner N. J. Pinggera A. (2015). Pharmacology of L-type calcium channels: novel drugs for old targets? *Curr. Mol. Pharmacol.* 8 110–122. 10.2174/1874467208666150507105845 25966690 PMC5384371

[B105] Takahashi J. S. (2017). Transcriptional architecture of the mammalian circadian clock. *Nat. Rev. Genet.* 18 164–179. 10.1038/nrg.2016.150 27990019 PMC5501165

[B106] Tanenhaus A. K. Zhang J. Yin J. C. (2012). In vivo circadian oscillation of dCREB2 and NF-kappaB activity in the *Drosophila* nervous system. *PLoS One* 7:e45130. 10.1371/journal.pone.0045130 23077489 PMC3471920

[B107] Tedesco D. Haragsim L. (2012). Cyclosporine: a review. *J. Transplant.* 2012:230386. 10.1155/2012/230386 PMC3259474 22263104

[B108] Videnovic A. Lazar A. S. Barker R. A. Overeem S. (2014). ‘The clocks that time us’–circadian rhythms in neurodegenerative disorders. *Nat. Rev. Neurol.* 10 683–693. 10.1038/nrneurol.2014.206 25385339 PMC4344830

[B109] Wang Q. Abruzzi K. C. Rosbash M. Rio D. C. (2018). Striking circadian neuron diversity and cycling of *Drosophila* alternative splicing. *Elife* 7:e35618. 10.7554/eLife.35618 PMC6025963 29863472

[B110] Welsh D. K. Takahashi J. S. Kay S. A. (2010). Suprachiasmatic nucleus: cell autonomy and network properties. *Annu. Rev. Physiol.* 72 551–577. 10.1146/annurev-physiol-021909-135919 20148688 PMC3758475

[B111] White W. B. Anders R. J. Macintyre J. M. Black H. R. Sica D. A. (1995). Nocturnal dosing of a novel delivery system of verapamil for systemic hypertension. Verapamil Study Group. *Am. J. Cardiol.* 76 375–380. 10.1016/s0002-9149(99)80104-0 7639163

[B112] Yamanaka N. Marques G. O’connor M. B. (2015). Vesicle-mediated steroid hormone secretion in *Drosophila melanogaster*. *Cell* 163 907–919. 10.1016/j.cell.2015.10.022 26544939 PMC4636736

[B113] Yokota S. Yamamoto M. Moriya T. Akiyama M. Fukunaga K. Miyamoto E. (2001). Involvement of calcium-calmodulin protein kinase but not mitogen-activated protein kinase in light-induced phase delays and Per gene expression in the suprachiasmatic nucleus of the hamster. *J. Neurochem.* 77 618–627. 10.1046/j.1471-4159.2001.00270.x 11299324

[B114] Zhang R. Lahens N. F. Ballance H. I. Hughes M. E. Hogenesch J. B. (2014). A circadian gene expression atlas in mammals: implications for biology and medicine. *Proc. Natl. Acad. Sci. U.S.A.* 111 16219–16224. 10.1073/pnas.1408886111 25349387 PMC4234565

[B115] Zhang X. Odom D. T. Koo S. H. Conkright M. D. Canettieri G. Best J. (2005). Genome-wide analysis of cAMP-response element binding protein occupancy, phosphorylation, and target gene activation in human tissues. *Proc. Natl. Acad. Sci. U.S.A.* 102 4459–4464. 10.1073/pnas.0501076102 15753290 PMC555478

